# Association of Serum Periostin with Cardiac Function and Short-Term Prognosis in Acute Myocardial Infarction Patients

**DOI:** 10.1371/journal.pone.0088755

**Published:** 2014-02-21

**Authors:** Lin Ling, Yan Cheng, Liucheng Ding, Xiangjun Yang

**Affiliations:** 1 Department of Cardiology, the First Affiliated Hospital of Soochow University, Suzhou, China; 2 Department of Cardiology, The Affiliated Wuxi People’s Hospital of Nanjing Medical University, Wuxi, China; 3 The Second Affiliated Hospital of Nanjing Medical University, Nanjing, China; University of Western Ontario, Canada

## Abstract

**Background:**

Periostin was proved to play an important role in extra-cellular matrix remodeling after acute myocardial infarction (AMI). Myocardial periostin was markedly up-regulated after AMI and participated in the maladaptive process of cardiac remodeling. However, few researches focused on the circulating periostin and its significance. This study aims to investigate the association of serum periostin level with cardiac function and short-term prognosis in AMI patients.

**Methodology/Principal Findings:**

We totally recruited 50 patients diagnosed as ST-elevation myocardial infarction. Blood samples were taken within 12 hours after the onset of AMI before emergency coronary revascularization procedures. Serum periostin was measured using enzyme-linked immunosorbent assay. All patients received echocardiography examination within one week after hospitalization. Correlations of serum periostin with echocardiography parameters, Killip class and myocardium injury biomarkers (CK-MB/troponin T) were investigated. AMI patients were divided into two groups by serum periostin level (higher/lower periostin group) and followed up for six months. Primary endpoints included cardiovascular mortality, nonfatal stroke/transient ischemic attack, chest pain occurrence and re-hospitalization. Secondary endpoint referred to composite cardiovascular events including all the primary endpoints.

**Result:**

Serum periostin was in negative association with left ventricular ejection fraction (LVEF) (r = −0.472, *p<0.01) and left atrium diameter (LAD) (r = −0.328, *p<0.05). Positive correlation was found between serum periostin level and Killip class (r = 0.395, *p<0.01). There was no association between serum periostin and CK-MB or troponin T (*p*>0.05). After six months follow up, patients in higher periostin group showed increased composite cardiovascular events (*p<0.05). Patients showed no significant difference in primary endpoints between the two groups.

**Conclusions/Significance:**

Serum periostin was in negative correlation with LVEF and LAD, in positive association with Killip class and higher serum periostin level may be predictive for worse short-term disease prognosis indicated as more composite cardiovascular events six months post AMI.

## Introduction

Acute myocardial infarction (AMI) remains as a worldwide public health problem with an increasing rate of incidence and causes irreversible cardiomyocytes impairment [1]. Ventricular remodeling due to persistent ischemia and hypoxia after myocardial infarction leads to the development of heart failure [2]. The interaction between myocytes and myocardial fibroblasts in the extra-cellular matrix (ECM) was proved to play a critical role in all stages of cardiac remodeling and subsequent heart failure. Activation of myocardial fibroblasts through both stretch-related signaling and neuron-endocrine effectors led to the secretion of various ECM proteins. These proteins conducted molecular signals, regulated cell kinetics and finally altered the structure of extra-cellular matrix [3], [4].

Periostin was a 90-kDa extra-cellular matrix protein secreted by fibroblasts, which acted as an important regulator in ventricular remodeling process such as pathological hypertrophy or fibrosis after myocardial injury. Periostin was proved to be related closely to the onset and development of coronary artery disease [5], [6]. Elevated expression of periostin protein was found in the infracted myocardium of AMI rat [7]. Exogenously over-expression of periostin gene in the heart led to impaired cardiac function, including left ventricle dilation, cardiac myocytes decrease and collagen deposition increase, which suggested a correlation between elevated periostin with deteriorated cardiac function. Furthermore, inhibition the expression of periostin was able to improve cardiac systolic ejection function and animal survival rate [8]. These researches demonstrated a detrimental effect of periostin in cardiovascular system. However, other researches have drawn opposite conclusions about the effect of periostin on myocardium remodeling. Dennis Ladage et al found that delivery of periostin peptide into the pericardial space of MI swine exerted beneficial effects on myocardium repair, reducing infarct myocardium size, attenuating left ventricular systolic function and increasing capillary density post MI. Another research group demonstrated that periostin was able to induce reentry of differentiated mammalian cardiomyocytes into the cell cycle both *in vitro* and *in vivo*. Delivery of recombinant periostin into the heart of MI rat improved cardiac systolic function, reduced fibrosis and hypertrophy as well as enhanced myocardium repair [9]–[10].

Although the net effect of periostin on ventricular remodeling after acute myocardial infarction has not reached a consensus, there is no doubt that this molecule acts as an important regulator in this process. Myocardial periostin was significantly up-regulated after AMI and participated actively in cardiac remodeling. However, few researches focused on the change in circulating periostin after AMI. This study was designed to investigate the association of serum periostin level with cardiac function and short term disease prognosis in acute myocardial infarction patients. Our study found that serum periostin was in negative association with left ventricular ejection fraction and left atrium diameter as well as in positive association with Killip class in acute myocardial infarction patients. Higher serum periostin level was related to increased composite cardiovascular events after six months follow up post AMI.

## Materials and Methods

### Ethics Statement

Clinical researches were approved by the Ethics Review Board for Clinical Studies of the First Affiliated Hospital of Soochow University (Suzhou, China, Approval Number: SZC10582). All clinical studies confirmed to ethics guidelines of the 1975 Declaration of Helsinki. All patients were informed with and signed written consent of this study. We did not conduct research outside of our country of residence.

### Study Subjects

We totally enrolled 50 patients of acute myocardial infarction (ST elevation) in our study. All patients were in consistent with the following diagnostic standards for ST-elevated acute myocardial infarction: i Clinical history of ischemic type chest pain; ii ECG changes. New ST elevation at the J point in at least 2 contiguous leads of 0.2 mV in men or 0.15 mV in women in leads V2–V3 and/or of 0.1 mV in other contiguous chest leads or the limb leads; New or presumably new left-bundle branch block; iii Evidence of myocardial injury or necrosis indicated by elevated serum cardiac biomarker, especially troponin T (Clinical Practice Guidelines on Management of STEMI, 2013). All patients received coronary revascularization procedures and standard drug therapy for coronary artery disease after hospitalization. Basic clinical characteristics including gender, age, coronary heart disease history, hypertension history, diabetes history, smoking history were collected and fasting blood sample were taken for measurement of glucose, cholesterol, high-density lipoprotein cholesterol (HDLc), low-density lipoprotein cholesterol (LDLc), triglyceride and renal function. Blood sample for periostin and myocardial injury biomarkers evaluation was taken within 12 hours after the onset of AMI before emergent coronary revascularization procedures. All blood tests were performed in the hospital’s laboratory.

### Determination of Serum Periostin Level

Serum periostin was measured using the DuoSet® ELISA kits (R&D Systems) according to manufacture’s instructions. Briefly, plates were coated with 100 µl of capture antibody and incubated overnight. After blocking with PBS containing 1%BSA overnight, 100 µl of diluted serum samples or recombinant periostin standards at different concentrations were added and incubated for 2 h at 37°C. Then 100 µl of detection antibody was incubated for 2 h at 37°C and streptavidin-HRP for 20 min. Plate washing were performed with 400 µl of washing buffer (0.05% Tween-20 in PBS) after every incubation for at least three times. Wells were incubated with 100 µl substrate solution for 20 min and stopped by adding 50 µl stop solution (2 N H2SO4). The optical density was measured at 450 nm and 570 nm using a micro-plate reader (Bio-Rad Laboratories, CA, USA) and the absorbance at 570 nm was subtracted from the absorbance at 450 nm. Each experiment was performed in duplicate wells and averaged for statistical analysis.

### Evaluation of Patients Cardiac Function

Trans-thoracic echocardiography was performed to assess cardiac function of acute myocardial infarction patients. All patients finished echocardiography examination within one week after hospitalization. A trained echo-cardiologist blinded to the experiment performed all the measurements with GE Vivid 7 system (GE Health Care, Netherlands). Left ventricular M-mode spectrum was performed in standard parasternal long-axis view to measure LV diameter at end cardiac diastole (LVDd) and systole (LVDs). The Teicholz method was used to calculate left-ventricular volumes and left-ventricular ejection fraction. Index including left ventricular posterior wall thickness (LVPWT), inter-ventricular septal thickness at diastole (IVSTd), left atrial diameter (LAD) and aorta dimension (AoD) were measured. All measurements were repeated for three consecutive pulsation cycles and averaged for statistical analysis.

### Patients Follow up Visit

Patients involved in this study were followed up for major cardiovascular events six months after the onset of acute myocardial infarction. We set primary and secondary clinical endpoints for evaluation of major cardiovascular events. Primary endpoints included cardiovascular mortality, nonfatal stroke or transient ischemic attack, typical chest pain occurrence and re-hospitalization due to unstable angina pectoris, re-infarction or heart failure. Cardiovascular mortality was defined as death due to fatal myocardial infarction, coronary intervention procedure, congestive heart failure, stroke, sudden cardiac death or other cardiovascular causes. Secondary endpoint was defined as composite cardiovascular events including all the four primary endpoints.

### Statistical Analysis

All continuous variables were expressed as mean ± SD. Comparisons between two groups were performed with unpaired Student’s t-test and comparisons among three or more groups were assessed by one-way ANOVA with Dunnett’s post-test. Categorical variables were expressed as percentages. Chi-square test was used for comparisons of categorical variables. Spearman correlation was used to analyze the relationship between serum periostin level and other parameters. A *p* value of <0.05 (two-tailed) was considered significance. All analyses were performed using SPSS 17.0 software (SPSS, Inc.).

The authors have full access to the data and take responsibility for its integrity. All authors have read and agreed to the manuscript as written.

## Results

### Correlation of Periostin Level with Clinical Characteristics in AMI Patients

The average age of patients was 63.30±12.78 years. AMI patients showed an average serum periositn level of 203.35±16.90 ng/ml. We analyzed the relationships of serum periostin level with patients’ clinical characteristics by spearman correlation. Baseline characteristics including gender, CHD history, hypertension, diabetes mellitus, smoking history, age, heart rate, triglyceride, cholesterol, HDLc, LDLc, venous glucose, creatinine and blood urea nitrogen (BUN) showed no correlations with serum periostin level (Table 1).

**Table 1:** Relationship between Clinical Characteristics and Serum Periostin Level in AMI Patients.

Parameters	Periostin (ng/ml) or Pearson correlation coefficient	P value
Categorical variables		
Gender		
Male	200.55±18.72	0.783
Female	211.33±38.56	
CHD history		
+	146.97±45.33	0.501
−	205.70±17.48	
Hypertension		
+	185.31±16.11	0.138
−	238.37±38.08	
Diabetes mellitus		
+	217.86±20.77	0.489
−	193.68±24.66	
Smoking		
+	197.41±25.69	0.768
−	207.65±22.78	
Continuous variables	r	
Age (yr)	−0.138	0.341
Heart Rate (bmp)	0.109	0.450
Triglycerid (mmol/L)	0.031	0.829
Cholesterol (mmol/L)	0.043	0.769
HDLc (mmol/L)	−0.190	0.186
LDLc (mmol/L)	0.126	0.382
Glucose (mmol/L)	0.124	0.393
Creatinine (umol/L)	−0.021	0.882
BUN (umol/L)	−0.186	0.195

All determinations were performed in the fasting state. CHD: coronary heart disease; HDLc: high density lipoprotein cholesterol; LDLc: low density lipoprotein cholesterol; BUN: blood urea nitrogen; Date presented as mean±SD; Spearman correlation was used to analyze the relationship between periostin level and variables.

### Association of Periostin Level with Coronary/Echocardiography Parameters and Myocardium Injury Biomarkers in AMI Patients

**Figure 1:**
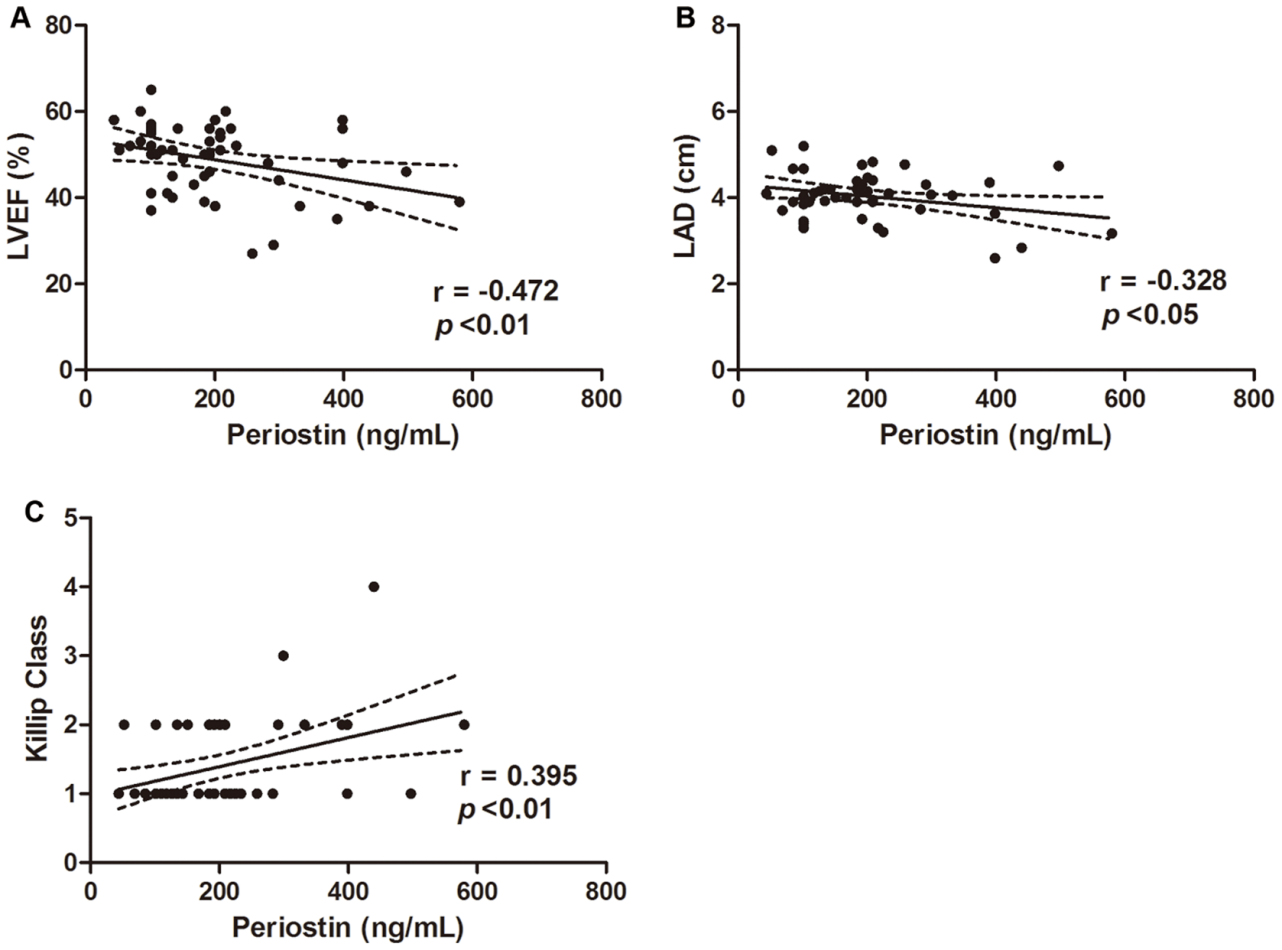
Association of periostin level with echocardiography parameters and Killip class. (A) Serum periostin level was in negative correlation with left ventricle ejection fraction in AMI patients (r = −0.472, *p*<0.01). (B) Serum periostin level was in negative correlation with left atrium diameter in AMI patients (r = −0.328, *p*<0.05). (C) Serum periostin level was in positive correlation with Killip class in AMI patients (r = 0.395, *p*<0.01).

**Figure 2:**
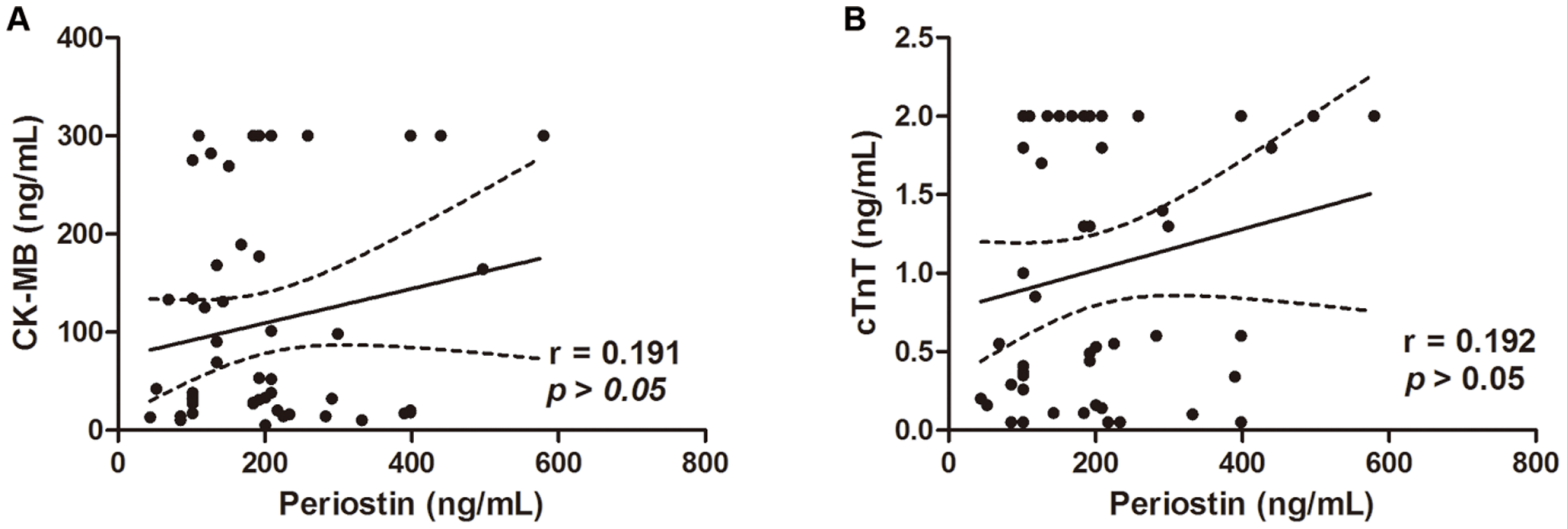
Serum periostin showed no correlation with myocardium injury biomarkers CK-MB (A) (r = 0.191, *p*>0.05) or Troponin T (B) (r = 0.192, *p*>0.05) in AMI patients.

Coronary and echocardiography parameters as well as myocardium injury biomarkers were assessed in AMI patients and their relationship with serum periostin level were analyzed. Number of diseased coronary vessels or implanted stents showed no correlation with periostin level. Among the echocardiography parameters, serum periostin was in negative correlation with left ventricular ejection fraction (r = −0.472, *p*<0.01) and left atrium diameter (r = −0.328, *p*<0.05) (Figure 1 A–B). Furthermore, serum periostin was in positive correlation with Killip class after AMI (r = 0.395, *p*<0.01) (Figure 1C). Patients classified as Killip I showed lower serum periostin level compared with those classified as Killip class III or higher (*p*<0.05). Correlation of periostin with coronary and echocardiography parameters was shown in Table 2. We also measured myocardial injury biomarkers in AMI patients. Serum troponin T and CK-MB showed good correlation with each other (r = 0.728, *p*<0.01) but neither of them showed significant correlation with serum periositn (Figure 2). Detailed parameters of echocardiography were presented in Table S1.

**Table 2:** Correlation of Periostin with Coronary/Echocardiography Parameters after AMI.

Parameters	Periostin (ng/ml) or Pearson correlation coefficient	P value
Number of Stents		
0 (n = 7)	173.47±35.64	0.947
1 (n = 23)	208.43±24.83	
2 (n = 12)	218.41±41.35	
3 (n = 5)	198.90±35.78	
CABG (n = 3)	181.32±109.80	
Number of Coronary Artery with Stenosis		
1 (n = 15)	196.15±34.74	0.552
2 (n = 17)	228.67±25.09	
≥3 (n = 18)	185.44±29.03	
Killip Class		
1	175.82±17.67	0.024*
2	241.76±35.05	
≥3	369.52±70.06	
Echocardiography parameters	r	
LVEF	−0.472	0.002*
LVDd	−0.163	0.285
LVPWT	−0.092	0.550
IVSTd	0.090	0.557
LAD	−0.328	0.028*
AoD	−0.114	0.456

LVEF: left ventricular systolic ejection fraction; LVDd: left ventricular end diastolic diameter; LVPWT: left ventricular posterior wall thickness; IVSTd: inter-ventricular septal thickness in diastole; LAD: left atrium diameter; AoD: aorta dimension. Date presented as mean±SD; Spearman correlation was used to analyze the relationship between periostin level and variables. *p<0.05.

### Patients with Higher Periostin Level Showed more Composite Cardiovascular Events after Six Months Follow up

**Figure 3:**
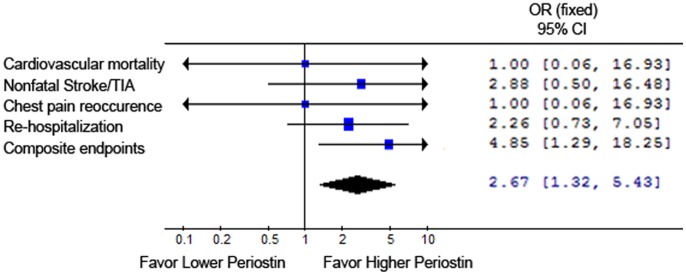
Effect of serum periostin on cardiovascular outcomes six months post AMI.

In order to assess the effect of serum periositn on short-term prognosis, we followed up patients for major cardiovascular events six months after the onset of AMI. All patients were divided into two groups based on median periostin level (188 ng/ml), named as higher periostin group (N = 25) and lower periostin group (N = 25). Basic clinical characteristics including gender, age, hypertension, diabetes, smoking history, blood glucose, cholesterol, HDLc, LDLc, triglyceride and renal function showed no difference between the two groups (Table 3). There was no difference in primary endpoints including cardiovascular mortality, nonfatal stroke or transient ischemic attacks, chest pain occurrence and re-hospitalization between the two groups. Patients with higher serum periostin showed increased composite cardiovascular events after six months follow up (*p*<0.05, Table 4). The net effect of perisotin on six month cardiovascular outcomes post-AMI was shown in Figure 3.

**Table 3:** Clinical Characteristics of AMI Patients at follow up.

Basic characteristics	Higher Periostin group	Low Periostin group	P value
N	25	25	–
Age (year)	64.2±12.02	62.4±13.69	0.624
Male (%)	80%	68%	0.333
Hypertension (%)	56%	76%	0.136
Diabetes mellitus (%)	36%	44%	0.546
Smoking (%)	52%	36%	0.254
Drugs	–	–	–
Aspirin	100%	100%	–
Clopidogrel	100%	100%	–
ACEI or ARB	100%	100%	–
Statins	100%	100%	–
triglyceride (mmol/L)	1.91±1.22	1.53±1.02	0.246
cholesterol (mmol/L)	4.58±1.07	4.14±0.97	0.139
HDLc (mmol/L)	0.87±0.2	0.98±0.41	0.230
LDLc (mmol/L)	2.50±0.76	2.08±0.79	0.065
Glucose (mmol/L)	7.21±3.13	6.96±2.41	0.751
Cr (umol/L)	67.79±15.75	71.14±22.26	0.542
BUN (umol/L)	6.01±1.71	6.46±2.21	0.414

All determinations were performed in the fasting state.

ACEI: Angiotensin-converting enzyme inhibitor; ARB: Angiotensin II receptor antagonists; HDLc: high density lipoprotein cholesterol; LDLc: low density lipoprotein cholesterol; Cr: creatinine; BUN: blood urea nitrogen.

Date presented as mean±SD.

**Table 4:** Cardiovascular Events of AMI Patients after Six Months Follow Up.

Cardiovascular Events	Higher Periostin group (N = 25)	Lower Periostin group (N = 25)	P value
**Primary endpoints**			
-Cardiovascular mortality	1	1	1.0
-Nonfatal Stroke/TIA	5	2	0.337
-Chest pain occurrence	14	9	0.156
-Re-hospitalization	1	1	1.0
**Secondary endpoints**			
-Composite endpoints	21	13	0.015*

TIA: transient ischemic attack; *p<0.05.

## Discussion

Acute myocardial infarction is one of the lethal diseases in the world, with an increasing morbidity and threatens the public health. The local ischemia and hypoxia conditions after AMI caused irreversible cardiomyocytes death or apoptosis and these cardiomyotes were gradually replaced by interstitial fibrosis through ventricular remodeling process. As scar and fibrosis structure lacked the ability of contractility and electric activity, systolic function and electric coupling of the heart were impaired and these finally led to deteriorated cardiac function [11], [12]. Activated fibroblasts in the extra-cellular matrix secreted various ECM proteins that contributed to ventricular remodeling after AMI. Through binding to targeted receptors and conducting cell signaling, these proteins played an important role in regulation of cardiomyoctes kinetics such as proliferation, migration and apoptosis [13]–[17].

Periostin is an extra-cellular protein proved to play an important role in cardiovascular development and disease [18]. Periostin is expressed at very early stages of embryogenesis but is not detectable in the normal adult heart. However, after cardiac injury such as myocardial infarction, pump failure or pressure overload, the mRNA and protein expression of periostin were greatly elevated in the heart [19]. Periostin showed close relationship with pressure overload induced maladaptive left ventricular hypertrophy, which was highly associated with end-stage heart failure [20]. In periostin knockout mice, cardiac healing after AMI was impaired and more cardiac ruptures were found. Smaller and less abundant collagen was also seen in periostin knockout mice, suggesting impaired collagen fibril formation in the lack of periostin gene [21]. Periostin over-expressed mensechymal stem cells implantation into the myocardium after AMI led to significantly histological and functional improvement, including decreased infarct size, reduced apoptotic myocardial cells and increased micro vessel density, indicating a cardio-protective role of periostin combined with stem cell transplantation [22]. Gene polymorphisms analysis in Chinese population showed that rs3829365 of the periostin gene was associated with susceptibility to, and severity of heart failure, suggesting a role of periostin in disease prediction and severity assessment [23]. Acute myocardial infarction patients showed decreased circulating level of periostin compared with control volunteers and periostin level was in negative correlation with patients’ cardiac function three months after AMI [24]. All these studies demonstrated that periostin acted as an important factor in the process of extracellular matrix remodeling under cardiac adverse conditions such as damage and pressure overload.

This study investigated the association of serum periostin level with cardiac function, coronary situation, biomarkers and clinical characteristics in acute myocardial infarction patients. The six month prognosis was also assessed. We found that serum periostin level was in negative association with left ventricular ejection fraction and left atrium diameter, in positive correlation with Killip class and showed no relationship with serum CK-MB or troponin T. Patients with higher serum periostin level exhibited increased composite cardiovascular events after six months follow up.

In our study, serum periositn level showed negative correlation with LVEF, suggesting that patients with lower left systolic function had higher circulating periostin level. Killip class represented left ventricular failure degree in the acute period after AMI. Serum periositn level was in positive correlation with Killip class. This was in consistent with the results of periostin and LVEF, suggesting a relationship of higher periostin level with worse left ventricular function. CK-MB and troponin T are well documented serum biomarkers for myocardial infarction and reflected myocardium injury [25]. We found no correlation between serum periositn and these two biomarkers, indicating that periositn was less likely an index related to myocardial injury. Whether higher circulating periostin level led to lower systolic function or resulted from worse heart function was not known. A lot of researches focused on the direct effect of periostin on cardiomyocytes and myocardium but had not reached a similar conclusion yet. Some showed that periostin was able to induce cardiomyocytes proliferation *in vitro* and acted as a cardio-protective factor [9]. Others found that over expression of periostin did not stimulate DNA synthesis, mitosis, or cytokinesis of cardiomyocytes in vitro or in vivo [26]. The actual effect of periostin, no matter it is protective, detrimental or neutral needs further confirmation.

According to our results, we concluded that periostin showed good correlation with cardiac systolic function and short term prognosis in ST elevation acute myocardial infarction patients. Higher serum periostin level was associated with worse left ventricular function and deteriorated short term prognosis. In the present study, we visited patients for six months and this was a relatively short period for myocardial infarction patients. Long term observation for cardiovascular major events and clinical prognosis would be needed in the further study. Our present study enrolled myocardial infarction patients with ST elevation and did not include non-ST elevation myocardial infarction patients or unstable angina patients. As these patients shared the similar pathological process as unstable plaque, the serum periostin level in these patients would better be analyzed in further experiments.

## Supporting Information

Table S1 Echocardiography Parameters of AMI Patients. LVEF: left ventricular systolic ejection fraction; LVDd: left ventricular end diastolic diameter; LVPWT: left ventricular posterior wall thickness; IVSTd: inter-ventricular septal thickness in diastole; LAD: left atrium diameter; AoD: aorta dimension. Date presented as mean±SD.
(DOC)
