# Supplementary material for: Association of Serum Periostin with Cardiac Function and Short-Term Prognosis in Acute Myocardial Infarction Patients
Source: PLoS One. 2014 Feb 21;9(2):e88755. doi: 10.1371/journal.pone.0088755 (PMC3931651; doi:10.1371/journal.pone.0088755)
Supplement: Table S1 — Echocardiography Parameters of AMI Patients. LVEF: left ventricular systolic ejection fraction; LVDd: left ventricular end diastolic diameter; LVPWT: left ventricular posterior wall thickness; IVSTd: inter-ventricular septal thickness in diastole; LAD: left atrium diameter; AoD: aorta dimension. Date presented as mean±SD. (DOC) [file pone.0088755.s001.doc]

**Supplementary Table 1**

**Echocardiography Parameters** **of AMI Patients**

| Echocardiography parameters | Higher Periostin group | Lower Periostin group | P value |
| --- | --- | --- | --- |
| N | 25 | 25 |  |
| LVEF (%) | 47.8±9.67 | 49.68±6.71 | 0.428 |
| LVDd (cm) | 5.36±0.62 | 5.32±0.49 | 0.797 |
| LVPWT (cm) | 0.89±0.17 | 0.88±0.10 | 0.743 |
| IVSTd (cm) | 0.91±0.16 | 0.87±0.19 | 0.397 |
| LAD (cm) | 4.06±0.48 | 3.99±0.59 | 0.692 |
| AoD (cm) | 3.22±0.28 | 3.12±0.27 | 0.188 |

LVEF: left ventricular systolic ejection fraction; LVDd: left ventricular end diastolic diameter; LVPWT: left ventricular posterior wall thickness; IVSTd : inter-ventricular septal thickness in diastole; LAD :left atrium diameter; AoD: aorta dimension. Date presented as mean±SD.
